# History of dermatology: the study of skin diseases over the centuries^☆^^☆☆^

**DOI:** 10.1016/j.abd.2020.09.006

**Published:** 2021-03-16

**Authors:** Iago Gonçalves Ferreira, Magda Blessmann Weber, Renan Rangel Bonamigo

**Affiliations:** a Universidade Federal de Ciências da Saúde de Porto Alegre, Porto Alegre, RS, Brazil; b Santa Casa de Misericórdia de Porto Alegre, Porto Alegre, RS, Brazil; c Faculty of Medicine, Universidade Federal do Rio Grande do Sul, Porto Alegre, RS, Brazil

**Keywords:** Dermatology, History, ancient, History, medieval, History of medicine, History, 18^th^ century, Skin diseases

## Abstract

The study of skin, the science of dermatology, has undergone significant transformations throughout the centuries. From the first descriptions of skin diseases in Egyptian papyri and in Hippocratic writings to the first treatises on dermatology, important individuals and discoveries have marked the specialty. In the 18^th^ and 19^th^ centuries, the specialty consolidated itself as a field of medical study based on the first classifications of dermatoses, diagnostic methods, and drug treatments. In the 20^th^ century, the scientific and technological revolution transformed dermatological practice, incorporating new therapeutic resources, as well as surgical and aesthetic procedures. In the face of such a vigorous process, it is important to provide a historical synthesis for the medical community to recognize and understand the origins that supported one of the most relevant specialties in the current medical scenario.

## Introduction

Skin disorders are a significant portion of the global total of diseases, affecting millions of people worldwide. Dermatology is the medical specialty responsible for the study of more than 4,000 diseases of skin and cutaneous adnexae, accounting for 15% to 30% of outpatient medical care in health systems, incorporating a wide arsenal of diagnostic, therapeutic, and aesthetic resources.^1^, ^2^, ^3^

Skin diseases have been known to mankind since its origin, considering that the essentially visual component of these conditions allowed their early recognition. The first records of cutaneous nosologies date back to ancient history, when they were described by the great civilizations that shaped Western medicine.^4^ From the Egyptian papyrus emerges the first skin hygiene measures, the handling of wounds, and the use of medicinal plants. From the postulates of Hippocrates, the father of medicine, physical inspection and clinical reasoning are established as pillars of medical diagnosis. Romans, Arabs, and Byzantines protected and contributed to the development of medicine for centuries, with marked advances in the light of the Renaissance and the Illuminism.^4^, ^5^

The study of skin diseases has been linked to general medicine for centuries. Only in the 18^th^ century, driven by the advancement of science and taxonomy in the fields of knowledge, did the first texts and works dedicated specifically to the study of skin diseases emerge.^4^, ^6^ From this pioneering period, the contributions of the great European schools of dermatology – Austrian, British, and French – stand out; through the discoveries, theories, classifications, and works of their renowned dermatologists, they allowed the consolidation of this important field of study and medical specialty.^7^, ^8^

Throughout the 19^th^ and 20^th^ centuries, the scientific revolution and technological innovations transformed dermatology, improving diagnostic techniques and providing new therapeutic resources. The specialty was consolidated through scientific societies, journals, and academic congresses, gradually attracting the interest of the medical community. In addition, dermatological practice expanded to encompass a wide range of surgical, diagnostic, and aesthetic procedures.^8^, ^9^, ^10^

Dermatology underwent an intense process of historical formation and transformation, following the evolution of contemporary medicine. The knowledge of the origins of the specialty, its great names, and its discoveries and works is an essential attribute for dermatologists and dermatology residents, as well as a valuable recognition for the medical community in general. Therefore, the article presents a theoretical review of the origins of dermatology, organized by the authors into five periods: primitive dermatology, pre-modern dermatology, modern dermatology, scientific dermatology, and technoscientific dermatology.

## Primitive dermatology: skin diseases in ancient times

### Ancient Egypt

Western medicine had its origins marked by two great ancient civilizations: Egyptian and Greek. Medical practice in Egypt was closely related to religion; priests provided medical care in religious temples, and diseases were attributed to the will of the gods. The manifestations of diseases and medical treatments were recorded on papyrus, a precursor of paper, in which clinical signs, diagnoses, plants, and therapeutic formulations were described.^5^, ^11^

**Figure 1:**
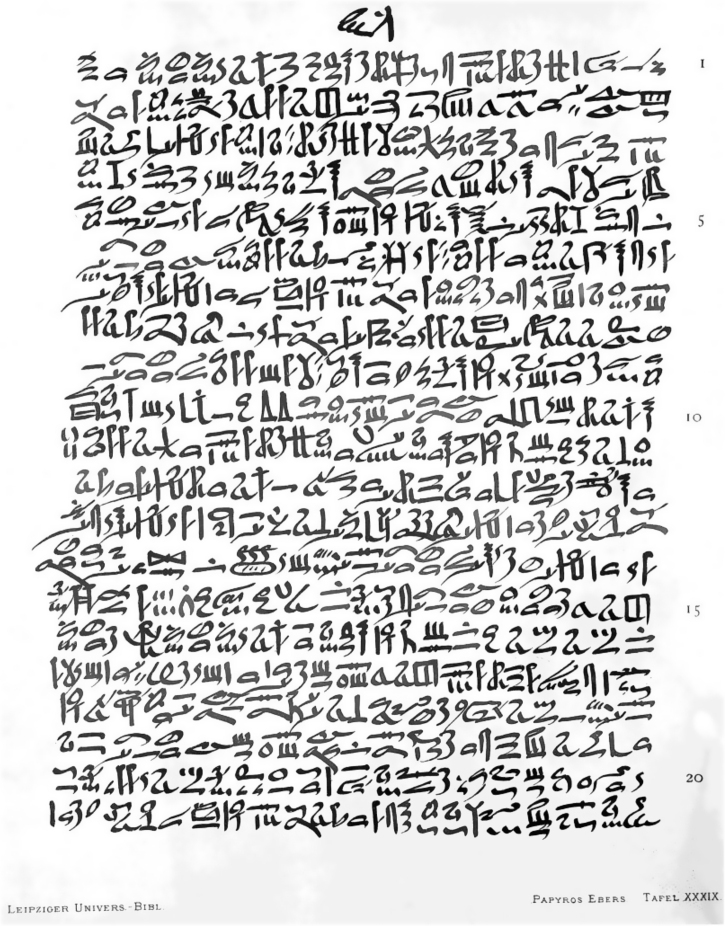
Ebers papyrus (1550 BC). Source: The Ebers papyrus – Wikimedia Commons.^13^

Skin diseases are mentioned in several texts, especially in the Edwin Smith (1600 BC) and Ebers (1550 BC) papyri (Fig. 1). The Edwin Smith papyrus, also known as Book of Wounds, has been recognized as one of the main medical texts of ancient Egypt, consisting of 48 cases of cutaneous injuries and wounds, many of which occurred during battles and accidents in Egyptian construction sites. This papyrus also addressed other medical fields such as general practice, gynecology, pediatrics, and even cosmiatry, mentioning rejuvenating prescriptions for skin.^5^, ^8^, ^11^, ^12^, ^13^

The Ebers papyrus is the largest medical document in ancient Egyptian medicine, with more than 800 chapters, covering various diseases such as urinary, digestive, and gynecological diseases, as well as skin and hair disorders, such as wounds, ulcers, and skin tumors. It also described approximately 1,500 magic and therapeutic formulas. Regarding tumor lesions, it addressed solitary tumors and those with “children” – probably referring to skin metastases. Similarly to the Smith papyrus, it also reinforced the aesthetic concern of the Egyptians, through the description of waxes and oils for the reduction of wrinkles and stains, and rudimentary chemical peeling techniques.^4^, ^5^, ^8^, ^14^

In addition, the Egyptians are credited with the rudimentary use of phototherapy, from the ingestion of the *Ammi majus L.* plant, found in the Nile delta, composed of methoxalene, a substance in the group of psoralens that acted as a natural photosensitizer. The use of this plant followed by sun exposure was included in the prescriptions of the Ebers papyrus, being recommended for repigmentation.^15^, ^16^

### Ancient Greece

In ancient Greece, Medicine and the art of healing were also attributed to divine entities. According to Greek mythology, Aesculapius was the god of medicine, and there were many temples dedicated to his worship on several Greek islands. In these temples, groups of priests called asclepiads treated diseases using medicinal plants, and even dog licking was used for the treatment of wounds.^4^, ^8^, ^17^

From the Greek island of Cos emerged one of the greatest exponents of Greek and Western medicine, the physician and professor Hippocrates (460–370 BC). Moving away from the metaphysical understanding of the health-disease process, Hippocrates contributed immensely to the rationalization of the clinical method, emphasizing clinical observation as an important resource for the diagnosis of diseases, thus becoming known worldwide as the father of modern medicine. In dermatology, a specialty in which observation is an essential attribute, the hippocratic contributions are also noteworthy.^4^, ^8^, ^18^, ^19^

Hippocrates proposed the first classification of skin diseases, dividing dermatoses into two classes: idiopathic diseases, which originate primarily from the skin, and exanthematic diseases (rashes), cutaneous manifestations of systemic diseases caused by imbalances in body humors (Table 1).^20^, ^21^

**Table 1:** Synthesis of classic works and the first classifications of dermatological diseases.

Author	Work	Models (criteria)	Classifications	
Hippocrates (460–370 B.C.)	*Corpus Hippocraticum* (IV – III BC)	2 classes (source)	Idiopathic diseases (originating from the skin)	
			Rashes (manifestations of systemic diseases): *Phymata* (furuncle and tumors); *Lopoi* (dry scaly eruptions); *Helchodia* (wet lesions)	
Girolamo Mercuriale (1530–1606)	*De Morbis Cutaneis*, *et Omnibus Corporis Humani Excrementis Ractatus Locupletissimi* (1572)	2 types (location)	Scalp diseases (*tineas*)	
			Diseases common to the entire skin (*tetter*): discoloration disorders; texture disorders; volume disorders	
Joseph Jacob Ritter von Plenck (1738–1807)	*Doctrina de Morbis Cutaneis* (1776)	14 classes (morphology, origin, and location)	Stains	Calluses
			Pustules	Excrescences
			Vesicles	Ulcers
			Blisters	Vulneras
			Papules	Insect bites
			Crusts	Nail disorders
			Scales	Hair diseases
Anne-Charles Lorry (1726–1783)	*Tractatus de Morbis Cutaneis* (1777)	2 classes (diseases – origin)	- Diseases originating from the skin	
			Diseases secondary to internal pathologies	
		6 types (injuries – morphology)	Simple pustules	
			Pustules containing a strange humor	
			Ulcers	
			Tumors	
			Patches	
			Scales	
Robert Willan (1757–1812)	*On Cutaneous Diseases* (1808)	8 orders (morphology)	Papules	Pustules
			Scales	Vesicles
			Rashes	Tubercles
			Blisters	Patches (dermal stains and growths)
Jean-Louis Alibert (1768–1837)	“*Description Des Maladies de la Peau Observées À L’hôpital St Louis*” (1806)	12 groups (morphology and origin)	Heteromorphic dermatoses	
			Eczematous dermatoses	
			Exanthematous dermatoses	
			Dyschromatous dermatoses (verify)	
			Mycological dermatoses	
			Variolous dermatoses	
			Desquamative dermatoses	
	“*Clinique de L’hôpital St Louis*” (1833)	51 types (source)	Haematological dermatoses	
			Cancerous dermatoses	
			Leprosy dermatoses	
			Scabious dermatoses	
			Foamy dermatoses	
Ferdinand von Hebra (1816–1880)	*Atlas der Hautkrankeiten* (1856)	12 classes	Hyperemia	
			Cutaneous anemia	
			Secretion disorders	
			Inflammation	
			Hemorrhage	
			Hypertrophies	
			Atrophies	
			Neoplasms (benign neoplasms)	
			Pseudoplasmas (malignant neoplasms)	
			Ulcerations	
			Neuroses	
			Parasites	

Source: Elaborated by the authors, based on the literature.^22^, ^29^, ^30^, ^31^, ^33^, ^38^, ^48^^,^^50^, ^51^

**Figure 2:**
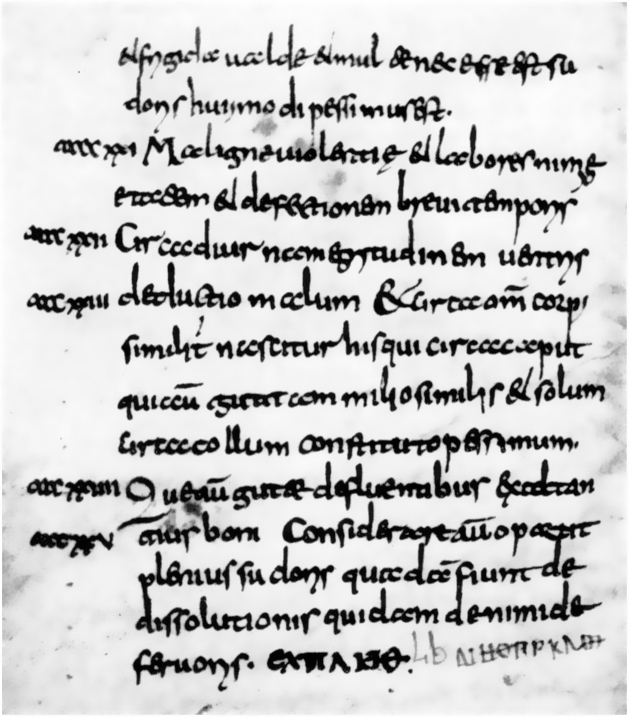
*Corpus Hippocraticum* (3^rd^ century BC). Source: Hippocratic Corpus – Wikimedia Commons.^23^

Approximately 100 years after his death, around the 3^rd^ century BC, the texts and theories of Hippocrates were brought together in the *Corpus Hippocraticum* (Fig. 2), one of the first historical milestones of medicine and dermatology. In that work, the anatomy and physiology of the skin were described in detail through its physiological processes, such as sweating and glandular secretion, as well as its role in the body's homeostasis. Based on the theory of the four bodily humors – blood, phlegm, black bile, and yellow bile – Hippocrates believed that dermatological diseases were manifestations of humoral imbalances.^4^, ^22^

The *Corpus Hippocraticum* presented descriptions of a wide variety of skin diseases such as acne, alopecia areata, and scaly, bullous, and pustular eruptions that, unlike the current nosologies, were believed to have a humoral etiology.^4^, ^7^, ^22^ Regarding treatments, the use of honey, tar, pig fat, and goose fat was recommended as topical therapy, due to the belief that the formulations should have an opposite effect to the pathological process: drying agents should be used in moist lesions and emollient lesions in dry lesions, principles still adopted by dermatology.^20^, ^22^, ^23^

### Roman Empire

In ancient Rome, hygiene and skin care occupied a relevant space in social life. Roman baths or public baths were intended for social gatherings and health care, in which the Romans bathed in pools of warm and cold waters, which were believed to have medicinal properties; they also cleaned the skin using instruments such as the strigil, a curved blade used for scraping. During bathing sessions, in addition to skin cleansing, body oils were applied in order to mitigate itchy disorders and moisturize the skin.^24^

Like the Egyptians, Romans believed that organs and diseases were associated with their gods, assigning each god a specific pathology. Under this logic, they attributed each disease to a specific doctor; therefore, some dedicated themselves to the treatment of hernias, eyes, ears, and skin, and the latter were responsible for the prescription of medicinal baths.^25^, ^26^

Despite the medical-religious approach, the process of applying science to Roman medicine began at the height of the Roman Empire. In this context, the contributions of Aurelius Cornelius Celsus (25 BC–50 AD) stand out. In his work *De Medicina*, a medical encyclopedia, Celsus dedicated an entire chapter to skin diseases, describing approximately 40 treatments for dermatological conditions.^24^, ^26^

The first descriptions of various skin lesions are attributed to Celsus, among them acrochordons, molluscum contagiosum, and kerion celsi, crusts on the scalp of children similar to honeycombs. In Greek, *kerion* means honeycomb, equivalent to the Latin term *favus*.^24^, ^26^

## Pre-modern dermatology: medieval inertia and the first classifications of skin diseases

During the Middle Ages, Europe experienced a long period of relative intellectual stagnation, in which superstitions and metaphysics dominated views about illness and medical care. Medicine was subordinated to the Catholic Church, which held the sources of knowledge, such as Greek medical texts, translated into Latin in medieval monasteries and churches.^4^, ^8^, ^25^, ^27^

The precarious living conditions and poor hygiene of medieval societies predisposed to the outbreak of several epidemics of infectious diseases, such as smallpox, which, when it did not cause death, generated debilitating sequelae such as skin scars, disfigurement, and alopecia. During this period, leprosy is estimated to have affected about 5% of the entire population of medieval Europe.^27^

In the 15^th^ and 16^th^ centuries, the Renaissance witnessed an intense expansion of knowledge and revaluation of Greek rationality, stimulating an increase in interest in the medical fields, including the study of the skin. Thus, the first classifications of dermatological diseases appear, as well as the expansion of notions of skin anatomy and topical therapies.^4^, ^6^

During this period, the pioneering work of Girolamo Mercuriale (1530–1606), professor of medicine at the University of Padova, Italy, stands out. Mercuriale proposed that dermatoses be classified into diseases of the scalp (*tineas*) and diseases common to other areas of the skin (*tetter*), the latter subdivided according to their morphological characteristics, color, texture, and volume (Table 1). Mercuriale is also recognized for his *De Morbis Cutaneis, et Omnibus Corporis Humani Excrementis Tractatus*, (Treatise of Diseases of the Skin and All Excrements of the Human Body) published in 1572 and considered the precursor of publications in dermatology.^28^, ^29^, ^30^, ^31^

**Figure 3:**
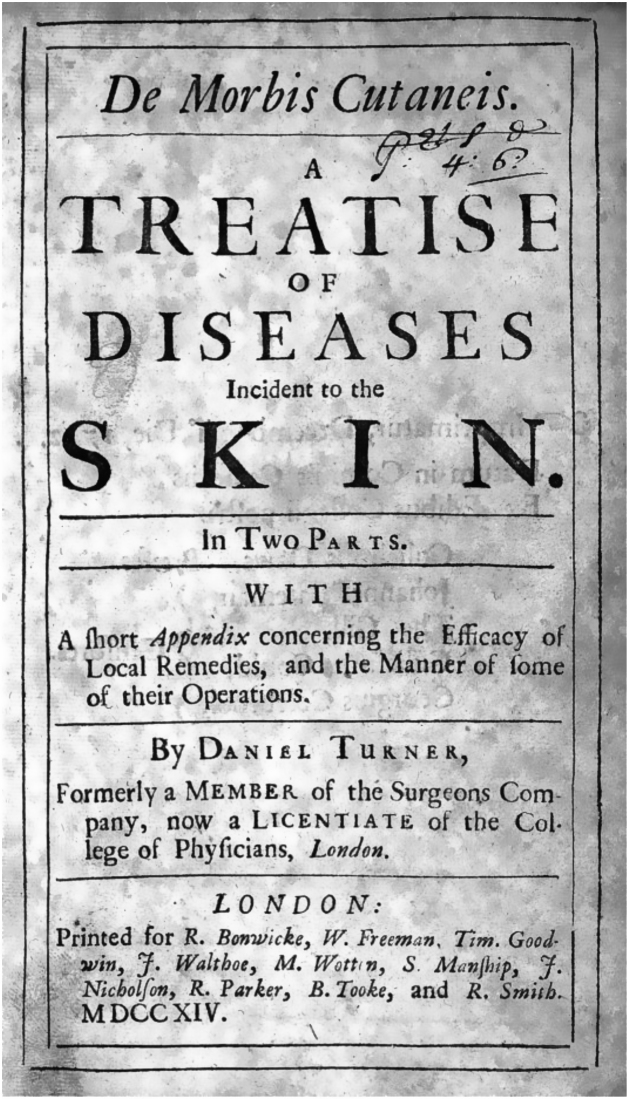
*De Morbis Cutaneis*, by Daniel Turner (1714) Source: *De Morbis Cutaneis*, London, 1714.^33^

Over a century after Mercuriale, Daniel Turner (1667–1741), through his work *De Morbis Cutaneis* (Fig. 3), introduces an interest in dermatology in the United Kingdom, being considered until the beginning of the 20^th^ century as the father of British dermatology. The treatise *De Morbis Cutaneis*, published in 1714, presented over 100 clinical cases of dermatological diseases and their respective treatments; it was translated into several languages and published in several editions.^7^, ^32^, ^33^

In the early 18^th^ century, the physician Jean Astruc (1684–1766) revealed the French pioneering spirit in dermatology. A physician at the court of King Louis XV, Astruc dedicated himself to the study of venereal diseases, publishing in 1736 the treatise *De Morbis Venereis*, a milestone in the history of venereology. Furthermore, he made significant contributions to the classification of rosacea, characterizing this condition in three forms: erythematous, varicose, and scaly.^34^, ^35^, ^36^

## Modern dermatology: the contributions of the great European medical schools

Throughout the 18^th^ and 19^th^ centuries, the study of dermatology intensified in Europe, with three major medical and research centers standing out: the United Kingdom, France, and Austria. Dermatologists from the British, French, and Austrian schools (Table 1, Table 2) established the foundations of the science of dermatology, providing discoveries, theories, and knowledge that still reverberate in the specialty.

**Table 2:** The great pioneers of dermatology and their contributions.

**Austrian School**	
Joseph Jacob Ritter von Plenck (1738–1807)	*Doctrina de Morbis Cutaneis* - classification of more than 150 dermatological diseases in 14 groups of dermatoses
	First to classify dermatoses by primary lesions
Ferdinand von Hebra (1816–1880)	First to classify dermatoses based on histopathological findings
	*Atlas der Hautkrankeiten* – the first atlas of dermatology in German
Moritz Kaposi (1837–1902)	First to describe idiopathic multiple pigmented sarcoma – Kaposi's sarcoma
	First to describe herpetic eczema, lupus erythematosus, and xeroderma pigmentosum
Heinrich Auspitz (1843–1878)	First to mention the terms "acanthoma" and "parakeratosis"
	Established the relationship between the papillary dermis and the epidermis in psoriasis lesions, corroborating the semiological maneuver known as Auspitz's sign
Paul Gerson Unna (1850–1929)	Description of histological structures of the skin and histopathological changes of skin lesions
	*Die Histopathologie der Hautkrankheiten –* first publication dedicated to cutaneous histopathology
	Invention of the bandage technique – Unna boot
**British School**	
Daniel Turner (1667–1741)	*De Morbis Cutaneis* – first book exclusively dedicated to skin diseases in English
	First series of cases in dermatology: reported more than 100 cases of skin diseases
Robert Willan (1757–1812)	*On Cutaneous Diseases* – 8 dermatoses orders, precise and organized definitions
	Classification of dermatoses by morphological characteristics and clinical signs of lesions
	Recognized as father of modern dermatology and British dermatology
Thomas Bateman (1778–1821)	*Practical Synopsis on Cutaneous Diseases* – continuation of the work of Robert Willan
	First to describe molluscum contagiosum, alopecia areata, and ecthyma
**French School**	
Jean Astruc (1684–1766)	*De Morbis Venereis* – first treatise on venereal diseases
Anne-Charles Lorry (1726–1783)	First to propose the concept of the skin as an organ
	*Tractatus de Morbis Cutaneis* - classification of skin diseases according to physiological, pathological, and etiological similarities
Jean-Louis Alibert (1768–1837)	Head of the first dermatology school/dermatological hospital (L'hôpital St Louis)
	Proposed the model known as the Tree of Dermatoses
Raymond Jacques Adrien Sabouraud (1864–1938)	Contributions to mycology in Dermatology - *Les Trichophyties Humaines*/*Les Teignes*
	Inventor of the culture medium Agar Sabouraud

Source: Elaborated by the authors, based on the literature.^30^, ^31^, ^32^, ^42^, ^43^, ^48^^,^^50^, ^51^, ^55^

### The Austrian School

The model of medical education integrated with hospital care, adopted by Central European countries, was a prosperous field for the development of new knowledge in the areas of bacteriology and histopathology, which contributed enormously to the understanding of the pathological processes of skin diseases.^8^

**Figure 4:**
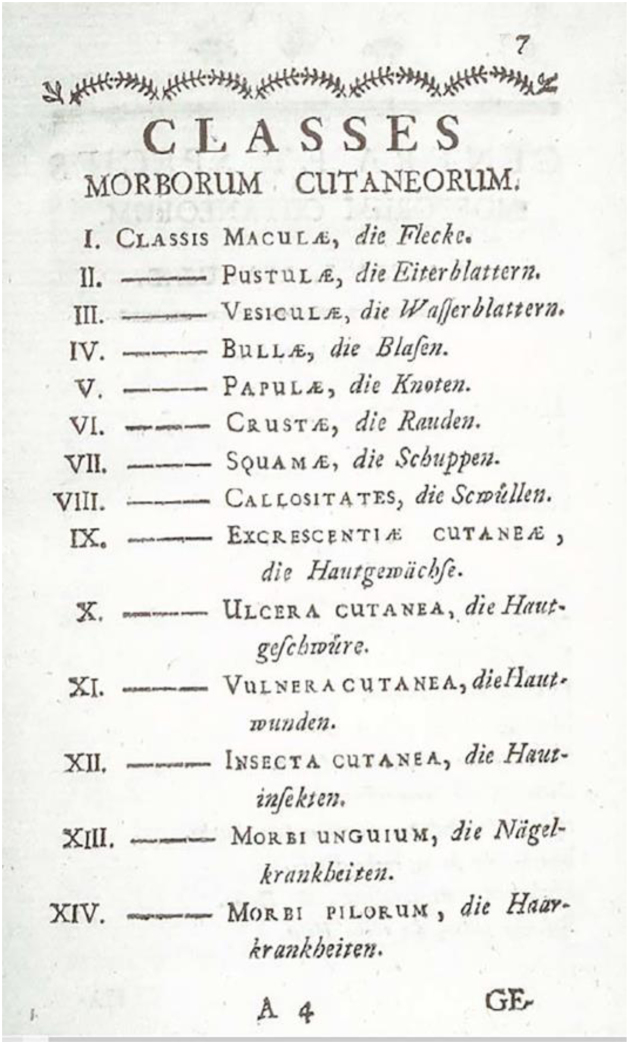
Classification of dermatological diseases by Plenck. Source: Doctrina de Morbis Cutaneis, Vienna, 1776.^38^

Born in Vienna, the Austrian physician Joseph Jacob Ritter von Plenck (1738–1807) devoted much of his life to the study of dermatological diseases. In 1776, he published the work *Doctrina de Morbis Cutaneis*, in which he described 150 types of skin diseases, categorized into 14 groups (Fig. 4); this was one of the first dermatological classifications to consider the morphological aspects of lesions.^8^, ^29^, ^30^, ^37^^,^^38^

Following Plenck's pioneering spirit, throughout the 19^th^ century the Vienna General Hospital (*Allgemeines Krankenhaus*) became an important center for research and teaching in dermatology, forming great names in the history of the specialty. Among its most famous dermatologists, the university professor Ferdinand von Hebra (1816–1880) deserves mention; he was notably recognized for his peculiar method of teaching dermatology that aroused the interest of several medical students.^8^, ^9^, ^39^

Throughout his professional career, Hebra produced numerous publications and monographs. However, he gained greater notoriety after the publication of the first atlas of dermatology in German – *Atlas der Hautkrankeiten* (1856) – which introduced the concepts of pathology to skin diseases, in addition to proposing a new classification of dermatoses based on anatomical and pathological findings.^4^, ^8^, ^9^, ^30^^,^^40^

For his valuable contributions, Hebra was recognized in Germanic countries as the founder of classical dermatology, attracting the interest of an entire generation of physicians. His disciples include Karl Rokitansky (1804–1878) and Carl Wedl (1815–1891), the originators of pathological anatomy; Moritz Kaposi (1837–1902), the first to describe Kaposi's sarcoma; and Heinrich Auspitz (1843–1878), the creator of the semiological maneuver known as Auspitz’s sign, which identifies psoriasis lesions.^4^, ^8^, ^9^, ^41^, ^42^, ^43^

German physician Paul Gerson Unna (1850–1929) was another notable dermatologist at the Austrian School, being considered one of the pioneers of modern dermatology. Unna studied clinical dermatology in Vienna, having the renowned dermatologists Hebra, Kaposi, and Auspitz as teachers. He returned to Hamburg in 1881, where he founded a private clinic that became a reference in training in dermatology, receiving apprentice doctors from different locations.^39^, ^44^, ^45^

**Figure 5:**
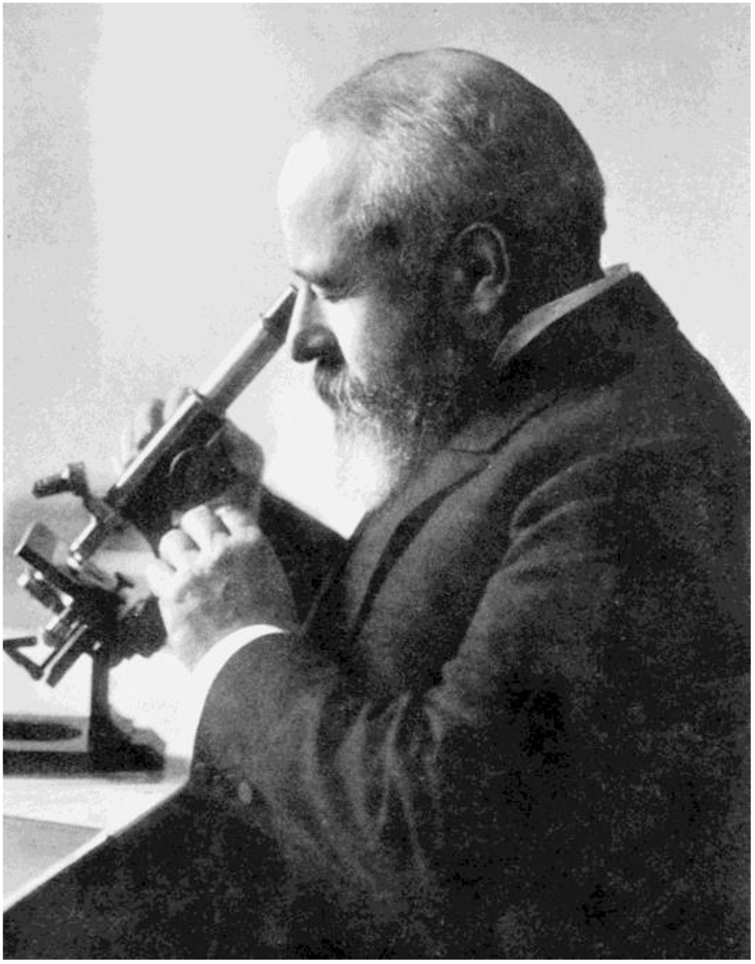
Paul Gerson Unna at his microscope, late 19^th^ century. Source: Paul Gerson Unna – U.S. National Library of Medicine.^46^

Through experiments in his clinic, Unna stood out for his discoveries on skin histology, recognizing collagen and elastic connective tissues, stratifying the epidermis and dermis layers, and defining the basal layer as a skin-regenerating layer. His studies in dermatological microscopy allowed the creation of new staining techniques and histological methods (Fig. 5). He was the first to describe histopathological changes such as acanthosis, spongiosis, and ballooning degeneration. In 1894, he became famous for publishing his findings in the book *Die Histopathologie der Hautkrankheiten* (The Histopathology of Skin Diseases), a milestone in the history of Dermatology.^37^, ^46^ Another of his relevant contributions was the invention of the bandage technique known as Unna boot, which to this day is used to treat venous stasis ulcers.^44^

### The British School

**Figure 6:**
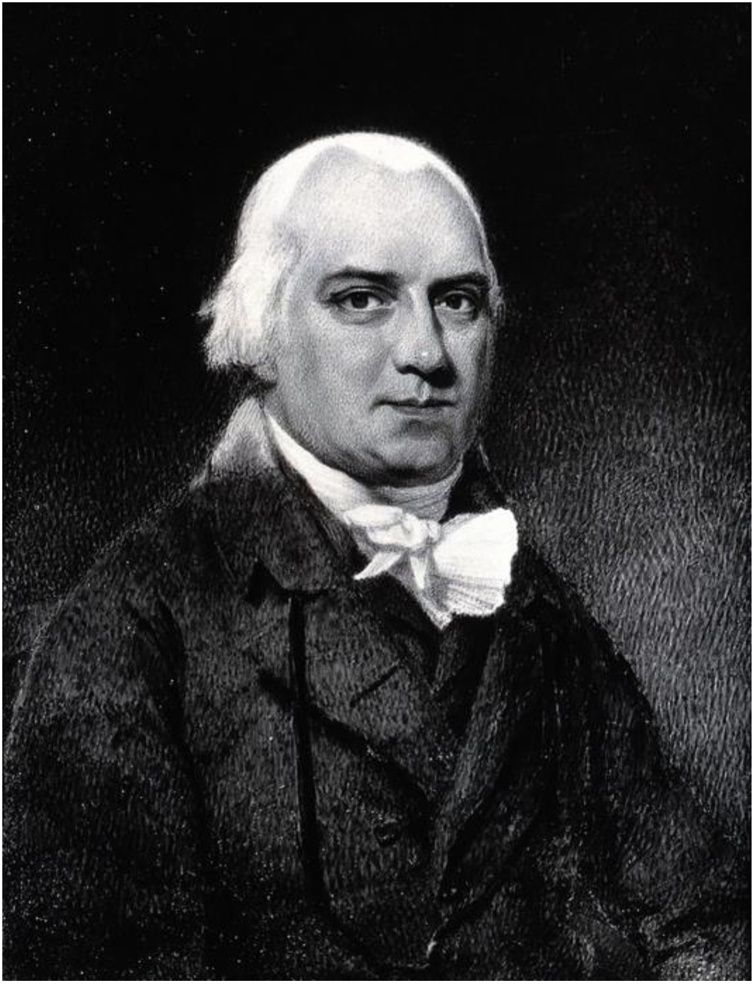
Robert Willan, the father of modern dermatology. Source: Wikimedia Commons – Robert Willan. Photograph by A. C. Cooper.^47^

After the pioneering work of Daniel Turner, British Dermatology was promoted again with the studies of Robert Willan (1757–1812) (Fig. 6). Willan worked as a physician at the Carey Street public dispensary and, from his observations in outpatient care, proposed a new approach to the classification of skin diseases, based on the morphological characteristics and clinical signs of the lesions.^7^, ^29^, ^47^

**Figure 7:**
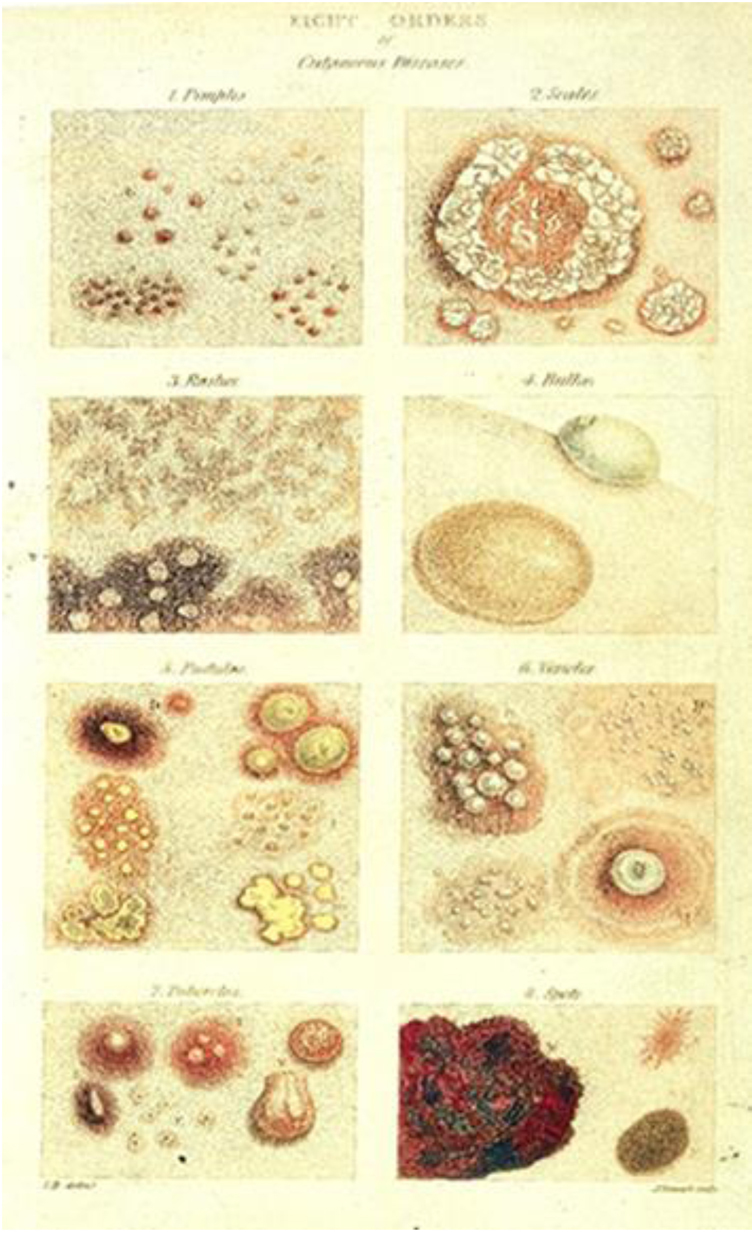
Illustrations of dermatological diseases by Robert Willan (1808). Source: On Cutaneous Diseases.^49^

Robert Willan described nine orders of dermatoses: papules, squamous, rashes, blisters, pustules, vesicles, tubercles, macules, and dermal outgrowths (Fig. 7). In 1808, Willan published the first volume of the work *On Cutaneous Diseases*, the first textbook of dermatology, in which he presented the first four orders of diseases: *Papulae* (papules), *Squamae* (scales), *Exanthemata* (rashes), and *Bullae* (blisters).^29^, ^37^, ^48^, ^49^ Robert Willan adopted precise and organized definitions for skin diseases, using terms adopted to this day to describe elementary lesions, for example. Unfortunately, his premature death prevented the completion of the second volume of the work, a task that was left to his apprentice Thomas Bateman.^4^, ^48^

Thomas Bateman (1778–1821) became the great broadcaster of Willan's work, publishing in 1813 the work *Synopsis of Cutaneous Diseases According to the Arrangement of Dr Willan*, a book with 11 editions translated into five languages.^7^, ^48^, ^50^ Robert Willan's contributions made him known as the founder of modern Western dermatology.^7^

### The French School

In the 18^th^ century, the study of dermatology was marked by two great works: the treatise *De Morbis Cutaneis* published by the British physician Daniel Turner in 1714, and the treatise *Tractatus de Morbis Cutaneis* (1777) by Anne-Charles Lorry, royal doctor of King Louis XVI. Jean Astruc's student, Anne-Charles Lorry (1726–1783) was one of the first dermatologists to understand the skin as an organ in itself, in interrelation with other organs.^29^, ^51^

In *Tractatus de Morbis Cutaneis*, a 700-page work written entirely in Latin, Lorry presented notions of skin anatomy, physiology, and pathology, and classified skin diseases according to physiological, pathological, and etiological similarities. Thus, he divided the dermatoses into primary, originating from the skin itself, and those secondary to internal diseases. In addition, he described six types of skin lesions: simple pustules, pustules containing a strange humor, ulcers, tumors, patches, and scales.^29^, ^51^, ^52^

**Figure 8:**
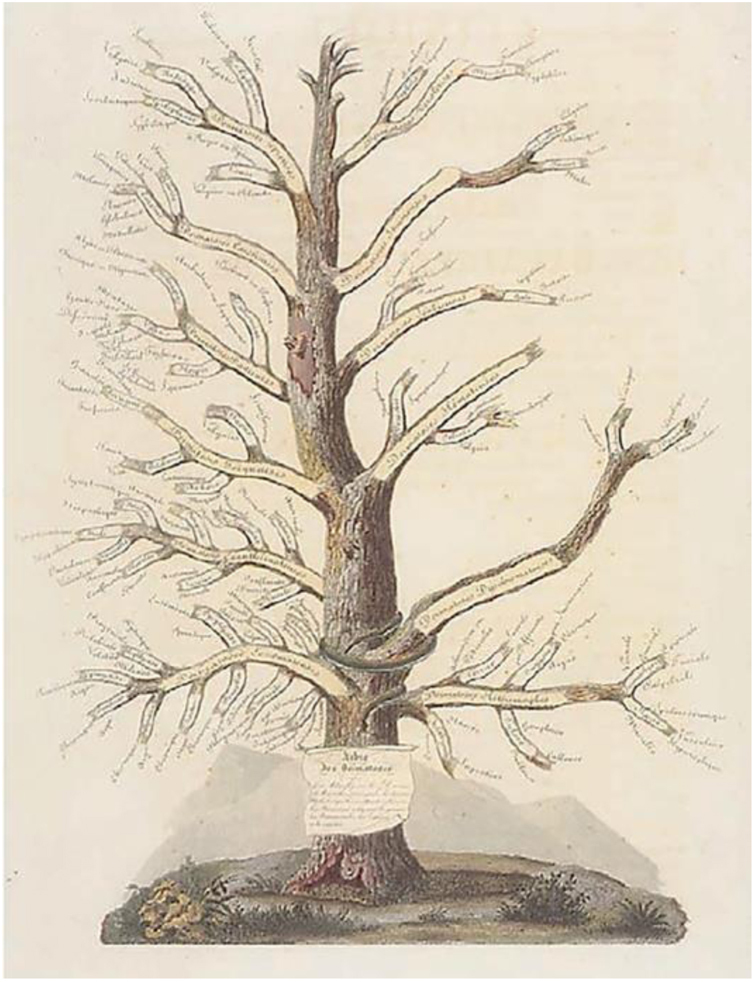
Jean-Louis Alibert's Tree of Dermatoses. Source: *Collection le Musée de L'hôpital St Louis*.^54^

At the beginning of the 19^th^ century, Paris was emerging as one of the great medical centers in Europe, primarily in the areas of clinical medicine and skin diseases.^9^ In this effervescent conjuncture, *L'hôpital Saint-Louis* stood out; one of the main medical centers in Paris, it was transformed into a dermatological hospital in 1801. Jean-Louis Alibert (1768–1837) was the first physician to dedicate himself to skin diseases at *L'hôpital Saint-Louis*; he was responsible for over 400 dermatological hospital beds. Based on the daily monitoring of the evolution of inpatients, Alibert outlined his first theories about skin diseases, classifying them into families, genera, and species, which formed the educational model known as the Tree of Dermatoses (Fig. 8).^4^, ^7^, ^8^, ^9^, ^53^, ^54^

Jean-Louis Alibert believed that skin diseases should be considered a part of internal medicine. In his atlases, skin disorders were pictured in persons, not just in the portion of lesional skin. Based on his experience, Alibert published the books *Description Des Maladies de la Peau Observées À L'hôpital Saint-Louis* (1806) and *Clinique de L'hôpital Saint-Louis* (1833), as well as the first mentions to mycosis fungoides, keloids, and cutaneous leishmaniasis, being recognized as the father of dermatology in France.^31^, ^53^

Alibert and Willan had significant divergences regarding their dermatological classifications and theories. The different approaches of the two dermatologists can be associated with the characteristics of the medical settings in which they were immersed: Willan performed his services in the outpatient clinics of the Carey Street dispensary, coming into contact with cutaneous lesions transversely, without the opportunity to monitor their evolution over the days. From another perspective, Alibert observed the dermatological conditions of his hospitalized patients on a daily basis, being able to more clearly identify the appearance, duration, and response to the adopted therapies.^7^

Dermatologist Raymond Jacques Adrien Sabouraud (1864–1938), another famous disciple of *L'hôpital Saint-Louis*, acquired notoriety for his studies in the field of mycology, in which he elucidated the etiological, pathological, and therapeutic aspects related to fungal skin diseases. In 1894, he reported his first research in mycology in the thesis *Les trichophyties humaines* (Human Trichophytoses). In 1910, he published the first complete treatise on mycology: *Les teignes* (Mycoses). In addition to his publications, Sabouraud has also become internationally recognized for the development of a standard culture medium for the isolation and identification of dermatophyte fungi, Sabouraud agar, composed of peptone, glucose, agar, and water.^37^, ^54^, ^55^

## Scientific dermatology: skin science and its discoveries

### The first scientific congresses and journals

**Figure 9:**
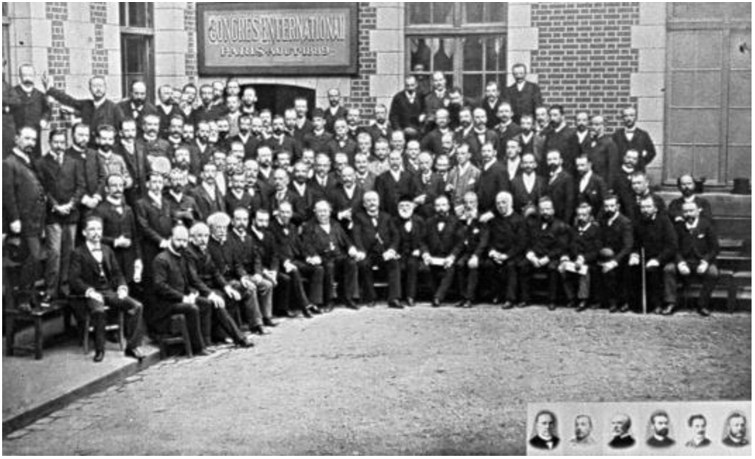
Dermatologists participating in the first International Congress of Dermatology and Syphilology – Paris (1889). Source: *Collection le Musée de L'hôpital St Louis*.^54^

The study of dermatology underwent a period of intense transformations and evolutions in the late 19^th^ century, becoming so extensive that the emergence of a specific and internationally consolidated medical specialty was inevitable.^8^, ^9^, ^10^ In this perspective, in 1889 the first International Congress of Dermatology and Syphilology was held in Paris, at the Dermatology Museum of St Louis Hospital (Fig. 9). The Congress had as honorary president the urologist Philippe Ricord (1800–1889), considered the father of French venereology in the 19^th^ century, and as the chairman the dermatologist Alfred Hardy (1811–1893), Alibert's former student and another disciple of the St Louis Hospital.^41^, ^56^

Aiming to promote knowledge of the specialty, the first specialized journals in Dermatology and Venereology were published in the 19^th^ century. The scientific journal *Syphilidologie,* founded in Leipzig in 1838, was the first publication of dermatological interest. In 1843, the journal *Annales des maladies de la peau et de la syphilis* was the first publication genuinely dedicated to dermatology.^41^

### Inventions that revolutionized dermatological practice

The expansion of scientific knowledge throughout the 20^th^ century and the development of new diagnostic and therapeutic resources led to several advances in dermatological practice. Some of the most important innovations of that period are highlighted:

#### Photography

Throughout the history of medicine, patients' clinical cases, diagnoses, and treatments were recorded in writing through notes and medical records, as well as shared among professionals through oral discussions and communications. However, images – fundamental for care and teaching in dermatology – relied exclusively on visual memory.^57^

The visual documentation of cutaneous diseases began with wood engravings, which evolved into woodcuts, multicolored copper engravings, and wax moulages, until reaching photographic records, with the invention of photography in 1840. Years later, in 1865, Alexander John Balmanno Squire (1836–1908) was the first dermatologist to apply photography to record skin diseases. Three years later, Alfred Louis Philippe Hardy, head of the dermatology department at L'h*ôpital Saint-Louis*, published the book *Clinique photographique de L'hôpital Saint-Louis*, a photographic series of the institution's dermatological clinical cases.^41^, ^57^ Since then, several atlases and textbooks have been published with image records of skin lesions, providing knowledge to several generations of dermatologists.

#### Dermatoscopy

The use of optical devices for the diagnosis of skin lesions started with surface microscopy, applied for the first time by Petrus Borrelius (1620–1671) in the observation of capillaries on the nail bed and folds, a technique currently known as capillaroscopy. In 1878, German physicist Ernst Karl Abbe (1840–1905) perfected the technique of using cedar oil as a means of contact in order to increase the sharpness of the images, a prototype of contact dermoscopy.^10^, ^58^

**Figure 10:**
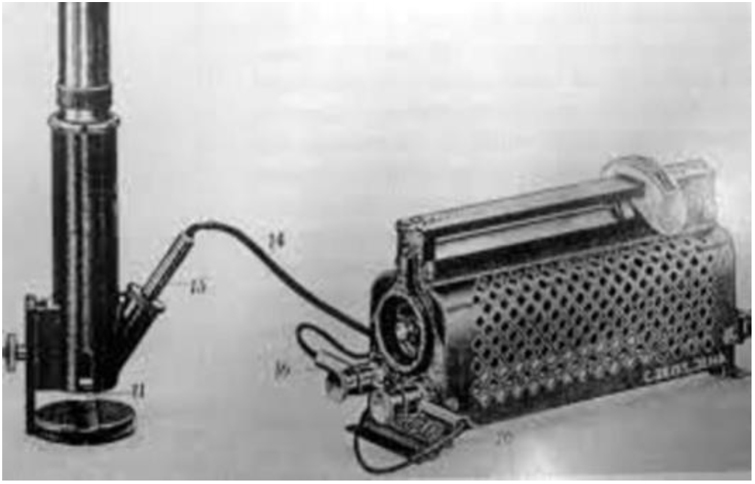
Surface microscope. Source: *História da Dermatoscopia*, Dominguez et al.^10^

The term dermoscopy was mentioned for the first time by Johan Saphier, in 1920, who described in his articles different applications of surface microscopy (Fig. 10), such as the study of capillaries, cutaneous tuberculosis, and syphilis lesions, and even melanocytic lesions and their globules, in which he still could not identify malignancy.^10^, ^58^, ^59^

The differentiation between benign and malignant melanocytic lesions was only described in 1971 by dermatologist Rona Mackie of the University of Glasgow, who reported the use of surface microscopy in the diagnosis of pigmented tumors.^10^, ^58^ Dermoscopy is currently considered essential for the diagnosis of skin tumors, as well as other conditions such as parasitic and inflammatory diseases.

#### Phototherapy

The first reports about sun exposure as a therapeutic resource in skin diseases date back to ancient Egypt, in which the plant *Ammi majus L.* was used as a photosensitizer in the treatment of vitiligo.^15^, ^16^ However, the first use of UV radiation to treat dermatological diseases is attributed to Niels Ryberg Finsen (1860–1904), a Danish physician who adopted filtered sunlight for the treatment of lupus vulgaris in 1893. Eight years later, in 1901, Finsen used the carbon arc lamp to treat lupus, winning the Nobel Prize for Medicine in 1903.^15^, ^60^

Broadband UVB radiation was introduced by William Henry Goeckerman (1884–1954) for the treatment of psoriasis in 1923.^15^ The 1960s and 1970s witnessed the first studies by dermatologist Thomas B. Fitzpatrick (1919–2003) on photochemotherapy with the use of psoralen (8-MOP), both topical and oral as photosensitizers, associated with high intensity UVA radiation, thereafter called PUVA (psoralen and UVA).^15^, ^60^

In 1981, Parrish and Jaenicke published a study in which they stated that the selection of wavelengths between 300 and 325 nm, as well as the use of monochromatic radiation instead of a broad band of radiation, would have a more effective therapeutic effect in the treatment of psoriasis, less damage to cellular DNA, and lower risk of erythema.^61^ Since then, narrowband UVB radiation has proved to be more effective, and became more widely adopted worldwide, being preferred over PUVA.^15^

#### Lasers

The use of lasers in dermatological treatments was pioneered by dermatologist Leon Goldman (1906-1997), who in 1963 reported the selective destruction of pigmented structures of the skin – nevi, melanomas, and tattoos – through the use of pulsed ruby laser. Later, in the book *Biomedical Aspects of the Laser* (1967), Goldman presents several possibilities and applications of lasers in medicine; in the following years, he reinforced these applications through the use of continuous-wave neodymium laser (Nd-YAG) in vascular malformations (1973) and in mixed cavernous hemangiomas (1976–1977).^16^, ^62^

In 1989, the Food and Drug Administration (FDA) approved the first laser to remove pigmented hair and in 1991, to remove tattoos.^16^, ^62^ Despite the initial lack of interest, lasers are currently gaining an important place in dermatology with a wide range of therapeutic and aesthetic applications.

#### Cryosurgery

British physician James Arnott (1797–1883) was the pioneer in the application of freezing techniques as a therapeutic resource. From 1845 to 1851, Arnott froze breast and skin tumors using saline solutions with crushed ice (-18 °C/-24 °C), noting the shrinkage of the lesions, as well as the analgesia provided by ice. In the following decades, other forms of freezing were developed, with emphasis on the use of freezing liquids and liquefied gases.^63^

The first use of liquid oxygen (-190 °C) in cryosurgery was performed in 1889 by dermatologist Campbell White, from New York, who used a cotton swab immersed in liquid oxygen and then applied it to skin lesions of herpes zoster, viral warts, lupus erythematosus, and basal cell carcinoma. Despite easy access to liquid oxygen, its combustive properties made its everyday use dangerous.^63^, ^64^

Safer, liquid nitrogen (-196 °C) was used clinically for the first time to treat viral warts, keratoses, and other non-neoplastic skin proliferations by physician Ray Allington.^63^, ^64^ In the 1970s, several cryosurgical devices with cryogenic agents emerged in addition to liquid nitrogen, including nitrous oxide, carbon dioxide, argon, ethyl chloride, and fluorinated hydrocarbons. Nowadays, cryosurgery is widely indicated for the treatment of benign and malignant skin lesions. ^63^

#### Dermatological surgery

Until the mid-20^th^ century, the practice of dermatology was essentially clinical, and most of the therapeutic modalities were restricted to the use of medications; skin biopsy was not a common resource and the excision of skin lesions was exceptional. In the 1950s and 1960s, dermatologists learned surgical procedures, developed techniques, publications, and technological resources that definitively transformed dermatology into a clinical-surgical specialty.^65^

**Figure 11:**
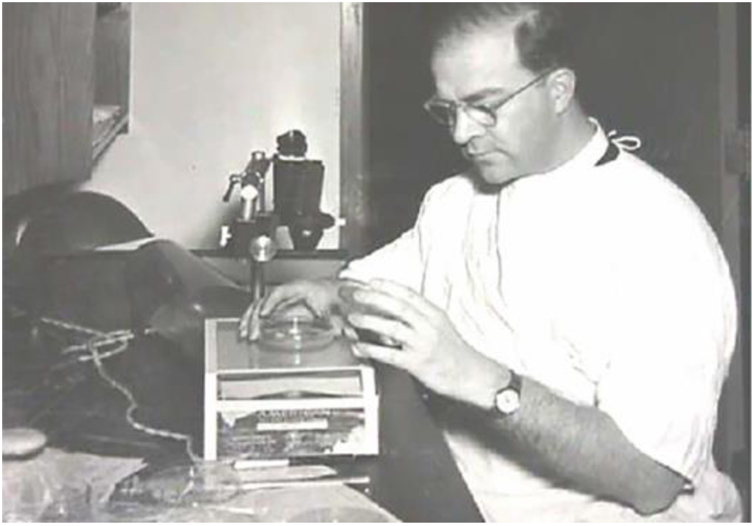
Frederic Edward Mohs - University of Wisconsin (1936*). *Frederic Mohs performing the chemosurgery procedure (micrographic surgery). Source: Skin Cancer Foundation.^69^

Among the advances in dermatology in the surgical area, the contributions of dermatologic surgeon Frederic Edward Mohs (1910–2002) at the University of Wisconsin are noteworthy. In 1940, Mohs described for the first time a surgical technique that allowed the excision of malignant neoplasms of the skin while preserving the healthy skin around the lesion, this technique was initially called chemosurgery with *in vivo* tissue fixation (Fig. 11). The term chemosurgery was adopted due to the use of zinc chloride for fixation. In 1956, when it was demonstrated the feasibility of analyzing small lesions in fresh tissue, he changed the nomenclature to microscopically controlled surgical excision.^65^, ^66^, ^67^, ^68^

The Mohs technique, widely termed Mohs micrographic surgery, consists of excising the skin cancer including both the affected tissue and the underlying tissue in depth, evaluating the parts excised under the microscope until a free margin is obtained. Mohs micrographic surgery was created and developed within dermatology, being recognized as one of the main milestones of the specialty.^65^, ^66^, ^67^, ^68^, ^69^

Another important milestone for dermatological surgery was the intumescent anesthesia technique, described in 1987 by the American dermatologist Jeffrey A. Klein (1944) at the University of California. The technique included subcutaneous infiltration of diluted lidocaine and epinephrine, causing edema and tumescence in the surgical area, which resulted in local anesthesia of the skin and subcutaneous tissue. Through this new technique, outpatient surgical procedures were widely adopted in dermatology, such as biopsies and excision of skin neoplasms, hair transplantation, and surgery for axillary hyperhidrosis.^65^, ^70^

#### Cosmiatry

The term cosmiatry was recognized by the medical community in 1959, during the World Congress of Dermatology in Stockholm (Sweden), thus gaining greater visibility among dermatologists.^71^ However, aesthetic procedures have aroused the interest of dermatologists for many centuries. In 1882, Paul Gerson Unna (1850–1929) presented in his publications the applications of desquamating agents such as phenol, trichloroacetic acid, resorcinol, and salicylic acid for chemical peels.^72^

From 1992 onwards, cosmiatry was intensely boosted in dermatology, since the description of the use of botulinum toxin for the treatment of expression wrinkles by the Carruthers, a husband-and-wife team (dermatologist and ophthalmologist).^65^ Botulinum toxin is a neurotoxin produced by the bacterium *Clostridium botulinum*, the etiological agent of botulism, a disease first reported in 1793 in Germany, after the death of several people due to food poisoning from eating uncooked sausages. For this episode, the German doctor Justinus Christian Kerner (1786–1862), the first to study botulism, called botulinum toxin the “sausage poison.” Emile Van Ermengem (1851–1932), a professor at the University of Ghent (Belgium), inspired by the idea of the “sausage poison” toxin, when isolating the bacteria that produced it in 1895, named it *Clostridium botulinum*, since *botulus* means sausage in Latin.^73^

During the 1990s, botulinum toxin acquired several clinical applications, such as in the treatment of strabismus, blepharospasm, hemifacial spasm, and cervical dystonia. However, it was only in 2002 that the Food and Drug Administration (FDA) approved its aesthetic use in the treatment of glabellar rhytids. Since then, the application of botulinum toxin has spread among dermatologists.^73^

In addition to botulinum toxin, other treatments for skin rejuvenation have been adopted by dermatologists, such as the use of fillers. In the late 1970s, Stanford University (United States) developed the first injectable dermal implant with bovine collagen (Zyderm I®), approved by the FDA in 1981 for filling soft tissues.^72^ However, because it is a heterologous substance, bovine collagen showed antigenic potential, with a short duration of effect.^74^

Synthetic fillers have demonstrated greater safety and longer duration. Hyaluronic acid, a linear polysaccharide composed of chains of glycosaminoglycans, has been recognized as the gold standard for the correction of facial rhytids, loss of contour, and replacement of facial volume. The first hyaluronic acid approved by the FDA for aesthetic use was Restylane®, in 2003, followed by several other forms in the following decades.^72^, ^74^

Another important filler, poly-l-lactic acid, commercially known as Sculptra® – a polymer of lactic acid molecules with a biostimulating action on collagen – was approved by the FDA for the first time in 2004, prescribed for the correction of HIV-associated lipodystrophy. Five years later it was authorized for use in aesthetic treatments, and is currently indicated for the improvement of skin flaccidity, volumetric correction, skin depressions, atrophic scars, and changes resulting from lipoatrophy.^75^, ^76^

## Technoscientific dermatology

Since the late 1990s and early 2000s, information and communication technologies (ICTs) have revolutionized social interactions and content sharing. As well as having an impact on forms of communication, these technologies can also contribute immensely to expanding access to health services and to fostering scientific research.^77^ In addition to ICTs, nanoscience – the study of particles on an atomic or molecular scale – has provided new perspectives on the use of substances and drugs on a nanoscale, providing greater specificity, half-life, and ability to penetrate tissues.^78^, ^79^ Thus, ICTs and nanoscience represent new perspectives relevant to dermatological practice.

### Teledermatology

Telemedicine is a technological resource that allows the transfer of health information for diagnostic, therapeutic, and continued education purposes.^79^ Telemedicine can have several applications in health services, such as: tele-appointments, telesurgery, tele-education, videoconferences, and telediagnosis.^80^

The essentially visual character of dermatological practice makes it a favorable field for telemedicine resources, known as teledermatology. Teledermatology presents several possibilities of application, such as long-distance dermatological consultations, screening of clinical cases referred by primary care, and educational activities such as training of residents, support for other specialties, and continuing medical education.^81^, ^82^

Among the possibilities of teledermatology, telediagnosis can be performed through the capture and transmission of digital photographs (macroscopy) and/or digital dermoscopy (with or without polarized light).^81^, ^82^

**Figure 12:**
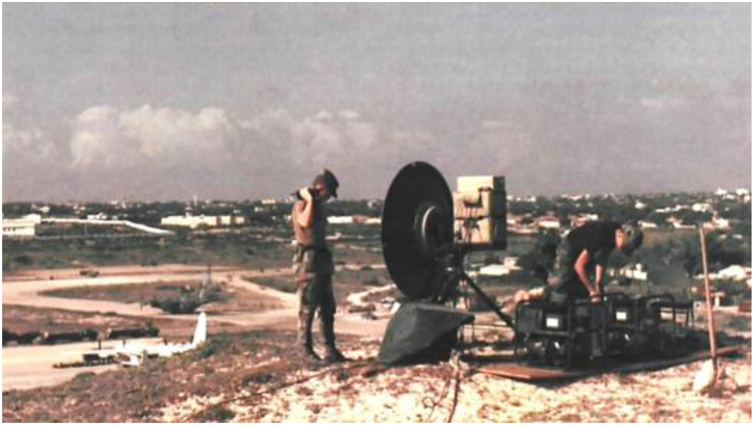
US Army satellite radio system in Somalia – Operation Restoring Hope (1992). Source: Restoring Hope – History Division – United States Marine Corps.^84^

The first experience using teledermatology took place in Somalia in 1992, during the United States Department of Defense's Restoring Hope mission. By capturing images of the combatants' skin lesions and transmitting them with the aid of a portable satellite radio (Fig. 12), the soldiers received dermatological assistance from North American dermatologists, thousands of kilometers away. In the following years, the United States Department of Defense expanded the services provided by the teledermatology service to other combat zones, using both asynchronous assistance, through image capture with subsequent dermatological evaluation, and videoconferences between combatants and dermatologists.^83^, ^84^

From these experiences, the literature and interest in teledermatology intensified through the 2000s and 2010s.^83^ With the expansion of access to wireless and mobile phone networks, the perspectives and research in the field of teledermatology indicate that this is an eminent field to be explored by dermatologists in the coming decades.

### Nanotechnology

Nanotechnology refers to particles smaller than 100 nm, equivalent to one billionth of a meter. Particles with such small dimensions demonstrate unique properties, which reveal different possibilities for the medical field.^85^ Physicist Richard Feynman (1918–1988) is considered the father of nanotechnology, due to his ideas regarding manipulating molecules and atoms in the 1950s. However, it was only in 1974 that the term nanotechnology was first mentioned by professor Norio Taniguchi (1912–1999) from the Tokyo University of Science.^78^

In dermatology, nanomedicine has raised significant interest of the cosmetic and pharmaceutical industries, in view of the pharmacological properties offered by this technology, allowing the increase in the bioavailability of substances in tissues, selectivity of action, and slow release; it also improves the texture and appearance of medications, photoprotectors, and emollients, among other topical therapies.^78^, ^85^

Despite its possibilities, nanomedicine presents risks that need to be better elucidated. These include the risk of toxicity caused by the greater availability of substance in the tissues, and the oncological potential, due to the deposition of nanoparticles in body tissues.^85^ Regardless of these controversies, nanomedicine undoubtedly still has a lot to contribute to dermatology.

## Final considerations

The study of the skin and its diseases has undergone intense transformations throughout its history, providing new knowledge, diagnoses, techniques, theories, classifications, and treatments, which have broadened the specialty's perspectives. From the prescriptions of the Ebers and Smith papyri, through the first dermatology treatises of Robert Willan and Jean-Louis Alibert, to the technological advances of the 20^th^ and 21^st^ centuries, important historical milestones for the science of dermatology occurred that deserve to be highlighted and recognized by anyone who intends to enter this vast and rich medical field.

## Financial support

None declared.

## Authors’ contributions

Iago Gonçalves Ferreira: Conception and/or design of the study; literature review and article selection; content analysis; results analysis; preliminary review and final drafting.

Magda Blessmann Weber: Conception and/or design of the study; literature review and article selection; content analysis; preliminary review and final drafting.

Renan Rangel Bonamigo: Conception and/or design of the study; literature review and article selection; content analysis; preliminary review and final drafting.

## Conflicts of interest

None declared.
